# The expression profiles of CD47 in the tumor microenvironment of salivary gland cancers: a next step in histology-driven immunotherapy

**DOI:** 10.1186/s12885-022-10114-4

**Published:** 2022-09-28

**Authors:** Michal Votava, Robin Bartolini, Linda Capkova, Jitka Smetanova, Vachtenheim Jiri, Martin Kuchar, David Kalfert, Jan Plzak, Jirina Bartunkova, Zuzana Strizova

**Affiliations:** 1 Department of Otorhinolaryngology and Head and Neck Surgery, First Faculty of Medicine, Charles University and University Hospital Motol, V Uvalu 84, 150 06 Prague 5, Czech Republic; 2 Chemokine Research Group, Institute of Infection, Immunity and Inflammation, College of Medical, Veterinary and Life Sciences, University of Glasgow, Glasgow, G12 8TT UK; 3 Department of Pathology and Molecular Medicine, Second Faculty of Medicine, Charles University and University Hospital Motol, V Uvalu 84, 150 06 Prague 5, Czech Republic; 4 Department of Immunology, Second Faculty of Medicine, Charles University and University Hospital Motol, V Uvalu 84, 150 06 Prague 5, Czech Republic; 5 Third Department of Surgery, 1st Faculty of Medicine, Charles University and University Hospital Motol, V Uvalu 84, 150 06 Prague 5, Czech Republic; 6 Department of Otorhinolaryngology and Head and Neck Surgery, University Hospital Bulovka, 18081 Prague, Czech Republic

**Keywords:** Oral Cancer, Immunotherapy, Tumor microenvironment, CD47, Don’t eat me signal, Mucoepidermoid carcinoma, Salivary gland cancer, Tumor-infiltrating lymphocytes, Metastatic SGC treatment

## Abstract

**Background:**

Salivary gland carcinomas (SGC) are extremely rare malignancies with only limited treatment options for the metastatic phase of the disease. Treatment with anti-CD47 antibodies could represent a potent therapy for SGCs by promoting the phagocytic clearance of tumor cells through various mechanisms. However, the efficacy of anti-CD47 therapy is largely dependent on the expression of CD47 within the tumor microenvironment (TME).

**Materials and Methods:**

In 43 patients with SGC, we were the first to investigate the CD47 expression in both tumor cells and tumor-infiltrating immune cells (TIIC) in the center and periphery of primary tumors. We also correlated the data with the clinicopathological variables of the patients and offered novel insights into the potential effectiveness of anti-CD47 therapy in SGCs.

**Results:**

We observed that the CD47^+^ tumor cells are outnumbered by CD47^+^ TIICs in mucoepidermoid carcinoma. In the tumor center, the proportion of CD47^+^ tumor cells was comparable to the proportion of CD47^+^ TIICs in most histological subtypes. In low-grade tumors, significantly higher expression of CD47 was observed in TIICs in the periphery of the tumor as compared to the center of the tumor.

**Conclusion:**

The reason for a high expression of ‘don’t eat me’ signals in TIICs in the tumor periphery is unclear. However, we hypothesize that in the tumor periphery, upregulation of CD47 in TIICs could be a mechanism to protect newly recruited leukocytes from macrophage-mediated phagocytosis, while also allowing the removal of old or exhausted leukocytes in the tumor center.

**Supplementary Information:**

The online version contains supplementary material available at 10.1186/s12885-022-10114-4.

## Background

CD47 is a transmembrane protein expressed in various cell types that belongs to the immunoglobulin superfamily [1]. CD47 binds to its ligands signal regulatory protein α (SIRPα), thrombospondin-1 (TSP-1), and integrins αvβ3 and α2β1 [2]. Specifically, SIRPα is highly expressed on the cell surface of macrophages, monocytes, neutrophils, and myeloid dendritic cells [3], and its interaction with CD47 was shown to potentiate 'don't eat me' signals that may inhibit macrophage-mediated phagocytosis [1, 3]. On the other hand, the CD47-SIRPα signaling pathway is involved in multiple signaling cascades and plays a crucial role in several physiological processes, such as phagocytosis of senescent erythrocytes or prevention of phagocytosis of circulating and tissue-resident immune cells [1, 4]. The CD47-SIRPα interaction is also involved in the organization of the secondary lymphoid organs and in the development of CD11c positive dendritic cells [5]. Several studies also indicate that CD47-SIRPα pathway may also lead to an inhibition of myosin which is required for phagocytosis [6, 7].

However, CD47 expression is also used by tumor cells to evade detection and phagocytosis by antigen-presenting cells, ultimately hampering the activation of the adaptive response and creating an environment that is permissive for tumor growth and spread [1].

CD47 is overexpressed in various cancer types, including breast cancer, acute myeloid leukemia (AML) and chronic myeloid leukemia (CML), leiomyosarcoma, and head and neck squamous cell carcinoma [8–11]. In multiple myeloma, CD47 expression was positively correlated with disease progression, while in ovarian carcinoma the low expression of CD47 in tumor cells not only reflected the patient's prognosis, but also predicted the patient's disease stage, and most importantly, the response to chemotherapy [12, 13].

For that reason, several anti-CD47 therapies have been developed to block CD47-SIRPα signaling pathway and promote macrophage phagocytosis of tumor cells [14].

Treatment with anti-CD47 antibodies can disrupt the interaction between CD47-SIRPα, allowing phagocytic clearance of tumor cells through various mechanisms: direct macrophage phagocytosis, antibody-dependent cellular cytotoxicity by natural killer cells (NK), phagocytosis and subsequent presentation of antigens by dendritic cells and / or induction of apoptosis [15–19].

Early successes in inducing tumor cell phagocytosis and inhibition of tumor growth *in vitro* and *in vivo* led to multiple preclinical and early clinical studies to assess the efficacy and safety of CD47-targeted anticancer therapies [20]. So far, several anti-CD47 antibodies, including Hu5F9-G4, CC-90002, and IBI188, have been tested in humans. In a phase 1b clinical trial in patients with invasive non-Hodgkin lymphoma, the addition of Hu5F9 to rituximab treatment was shown promising in inducing the antitumor activity in patients with rituximab-resistant disease [21]. Furthermore, two SIRPα-Fc fusion proteins, TTI-621 and ALX148, are being evaluated in phase I clinical trials [1].

Although preclinical models of CD47-based anticancer therapies are promising, many unknowns still surround this immunotherapeutic approach. Treatment response largely differs between cancer types and individuals, and this variation could be, in part, the result of differences in CD47 expression between diverse types of cancer [22–25]. In addition, the optimal dose and/or frequency of administrations must be carefully considered in each patient to achieve an effective therapeutic blockade of CD47 [25].

Salivary gland carcinomas (SGC) are extremely rare malignancies with 22 different histological subtypes [26]. The most prevalent SGC subtypes include adenoid cystic carcinoma (AdCC), acinic cell carcinoma (ACC), mucoepidermoid carcinoma (MEC), and salivary duct carcinoma (SDC), and their biological behavior, together with the prognosis of the patient, can vary substantially between subtypes [26, 27].

Treatment of SGCs is mainly surgical, and the response to chemotherapy is less than 30%, despite chemotherapy being one of the main options for metastatic disease [26, 28].

Currently, there are no treatment guidelines for metastatic SGC. In part, this is due to the rarity of these tumors. However, the extreme diversity of the SGC subtypes adds an additional layer of complexity, as the different subtypes respond differently to various therapies [27, 28].

Hence, molecular characterization of each SGC subtype is crucial to allow subtype-specific targeted therapy [28]. While several potential targets for systemic therapy have been identified so far, none have been evaluated in a randomized clinical trial to date [27].

Although different molecular patterns, such as Her-2 or PD-1/PD-L1 expression, have been studied in selected SGCs, so far the characterization of CD47 expression status in SGCs has not been carried out so far [29, 30].

We conducted a retrospective study investigating the expression of the CD47 molecule in both tumor cells and tumor-infiltrating immune cells (TIIC) in the center and periphery of primary tumors from 43 patients with SGC with 5 different histological subtypes. The data obtained was then matched with the clinicopathological data of the patients, such as tumor grade and stage.

To our knowledge, this is the first detailed analysis of CD47 expression in both tumor cells and SGC TIICs, and it offers insights into the potential effectiveness of anti-CD47 therapy as a treatment for these tumors.

## Materials and Methods

### Study cohort

To analyze intratumoral CD47 expression and clinicopathological characteristics of SGC, all patients who had undergone surgery between January 2014 and December 2021 for the diagnosis of MEC (13 patients), AdCC (10 patients), ACC (8 patients), SDC (6 patients) and adenocarcinoma not otherwise specified (AdCaNOS), (6 patients) were evaluated.

A total of 43 patients were included in the study. The tissue samples were retrospectively retrieved and the study was approved by the Ethics Committee of Motol University Hospital (EK-1394/20). The study was carried out according to the Declaration of Helsinki of 1964 and its subsequent amendments.

The patients were divided according to histology, tumor grade, and tumor stage. The female:male ratio was 25:16. The mean age of our patients was 59.3 years and the age ranged from 21 to 80 years.

### Immunohistochemical analysis

**Fig. 1.**
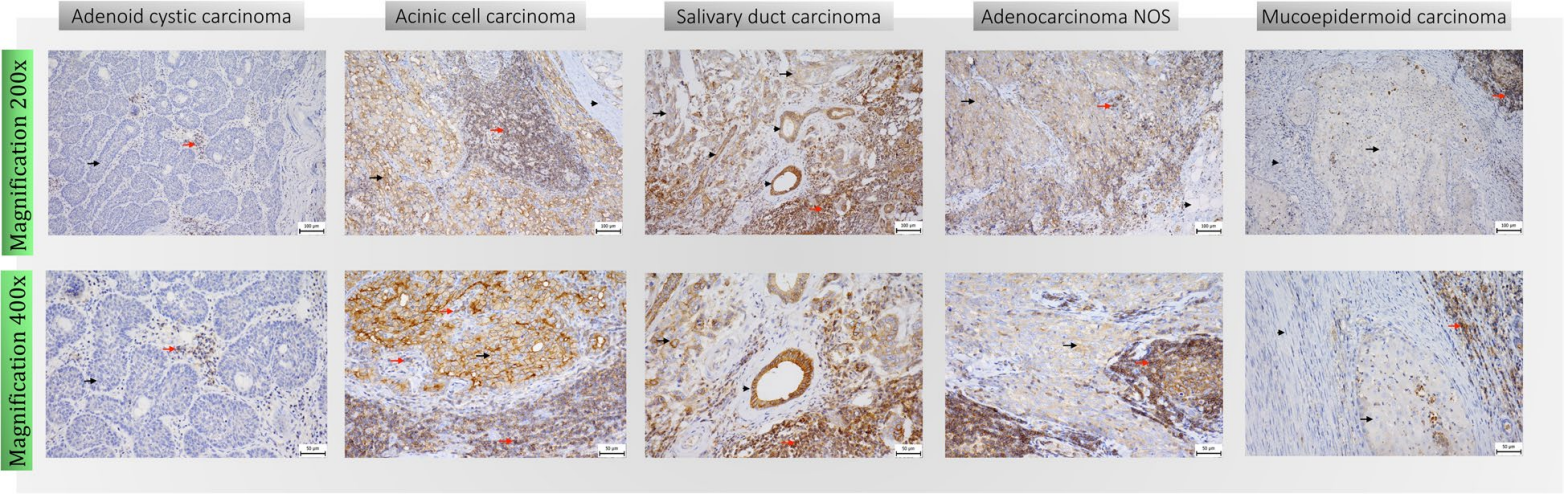
Immunohistochemistry. Immunohistochemistry (IHC) of CD47 expression in SGC tissues. Upper row shows 200x magnification images, lower row shows 400x magnification images. Representative images show CD47 expression with negative score in tumor cells (black arrow), and score 2 positivity in tumor-infiltrating immune cells (red arrow) in adenoid cystic carcinoma; CD47 expression with score 3 positivity in tumor cells (black arrow), score 3 positivity in tumor-infiltrating immune cells (red arrow) and negativity in stroma (black arrowhead) in acinic cell carcinoma; CD47 expression with score 2 positivity in tumor cells (black arrow), score 3 positivity in tumor-infiltrating immune cells (red arrow) and positivity in ductal epithelium (black arrowhead) in salivary duct carcinoma; CD47 expression with score 2 positivity in tumor cells (black arrow), score 3 positivity in tumor-infiltrating immune cells (red arrow) and negativity in adipose tissue (black arrowhead) in adenocarcinoma NOS; CD47 expression in tumor cells (black arrow) and tumor-infiltrating immune cells (red arrow) in mucoepidermoid carcinoma

Formalin-fixed paraffin embedded tissue samples (FFPE) were retrospectively recovered and stained for the presence of the CD47 molecule. Anti-CD47 antibody (PA5-80435, Thermofisher Scientific, Massachusetts, USA) was used for the detection of CD47 in tumor cells and tumor-infiltrating immune cells. From each patient, one resection tissue block of an overall area of 1cm2 was examined, with a minimum of 25 high-power field (HPF) of tumor area. To provide a complex analysis with respect to the peritumoral area, both the center and the periphery of the tumor were analyzed. The tumor-host interface that extends a HPF ( 400-fold magnification) from the tumor edge was considered as the tumor periphery. Each slide was manually scored by an experienced pathologist. Immune cells were identified by morphology in hematoxylin-eosin stain and the quality of the manual scoring was assessed by the intrarater reliability test to avoid subjective bias. For further verification, a subset of 10 cases was stained with CD3, CD4, CD8 and CD68 to specify the phenotypes of the infiltrating immune cells. Membranous and cytoplasmic staining was scored as positive in TIIC, and strong membranous staining was scored as positive in tumor cells [31, 32]. The scoring system was based on the percentage of tumor area covered by positive-stained cells [33–36]. The scoring was as follows: score 0 (no stain), negative; score 1 (weak staining), 1–10%; score 2 (moderate staining), 10–49%; and score 3 (strong staining), above 50%. Representative tissue samples are shown in Fig. 1. The CD47 staining on the adjacent normal tissue is shown in Supplementary Fig. 1.

### Statistical analysis

For statistical analyzes, GraphPad Prism 6 software (GraphPad Software, La Jolla, CA) was used. *P*<0.05 was considered significant. The indicated sample size (*n*) was used to calculate the means ± SEMs. The bivariate associations between the variables under study were evaluated using the Spearman rank order correlation coefficient. Differences in paired measurements were evaluated using the Wilcoxon signed rank test using a Monte Carlo resampling approach. P values were determined by the indicated test (*P<0.05, **P<0.01, ***P<0.001, and ****P<0.0001). *P*<0.05 was considered significant.

## Results

### Heterogeneous expression of CD47 was observed in tumor cells and tumor-infiltrating immune cells in the periphery NOS adenocarcinoma

**Fig. 2:**
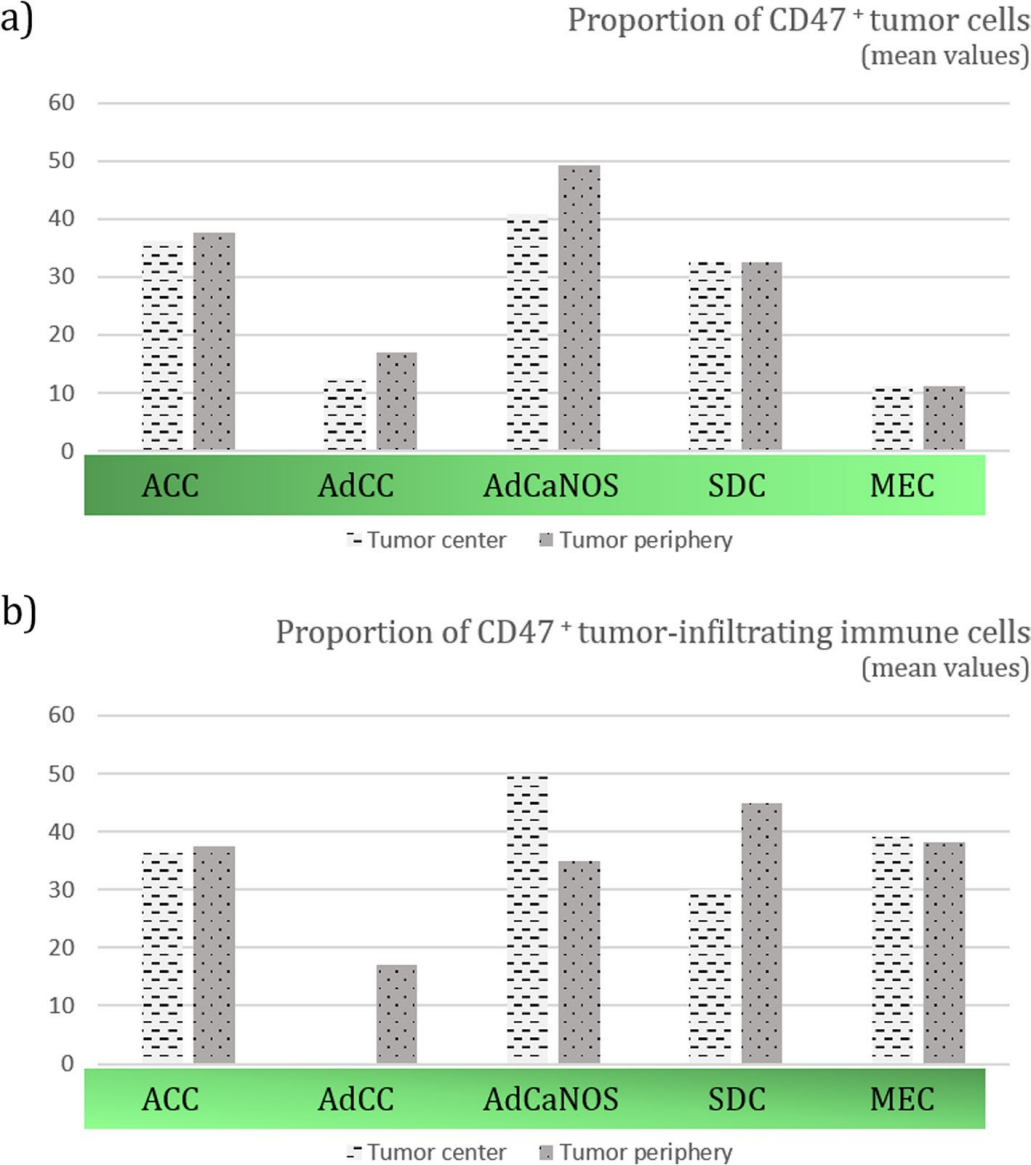
Proportions (%) of CD47 positive cells. Thirteen patients with mucoepidermoid carcinoma (MEC), ten patients with adenoid cystic carcinoma (AdCC), eight patients with acinic cell carcinoma (ACC), six patients with salivary duct carcinoma (SDC) and six patients with adenocarcinoma not otherwise specified (AdCaNOS) were included. The expression of CD47 differed between tumor cells (a) and TIICs (b) among histological subtypes and tissue compartments, such as the tumor center and tumor periphery. Mucoepidermoid carcinoma was shown to have the lowest infiltration with CD47^+^ tumor cells (2.a)

In our study, we evaluated 43 patients with the most prevalent histological subtypes of SGC. As high expression of CD47 in tumor cells was previously described as a negative prognostic factor in multiple types of cancer, such as breast cancer and ovarian cancer, we first attempted to analyze whether the expression of CD47 varies among histological subtypes of SGC. We observed that the highest infiltration with CD47 positive (CD47^+^) tumor cells was in the tumor periphery of AdCaNOS. Among all histological subtypes tested, the lowest infiltration with CD47 positive tumor cells was observed in MEC tumors (mean 11.25% of CD47^+^ tumor cells), Fig. 2a.

**Fig. 3:**
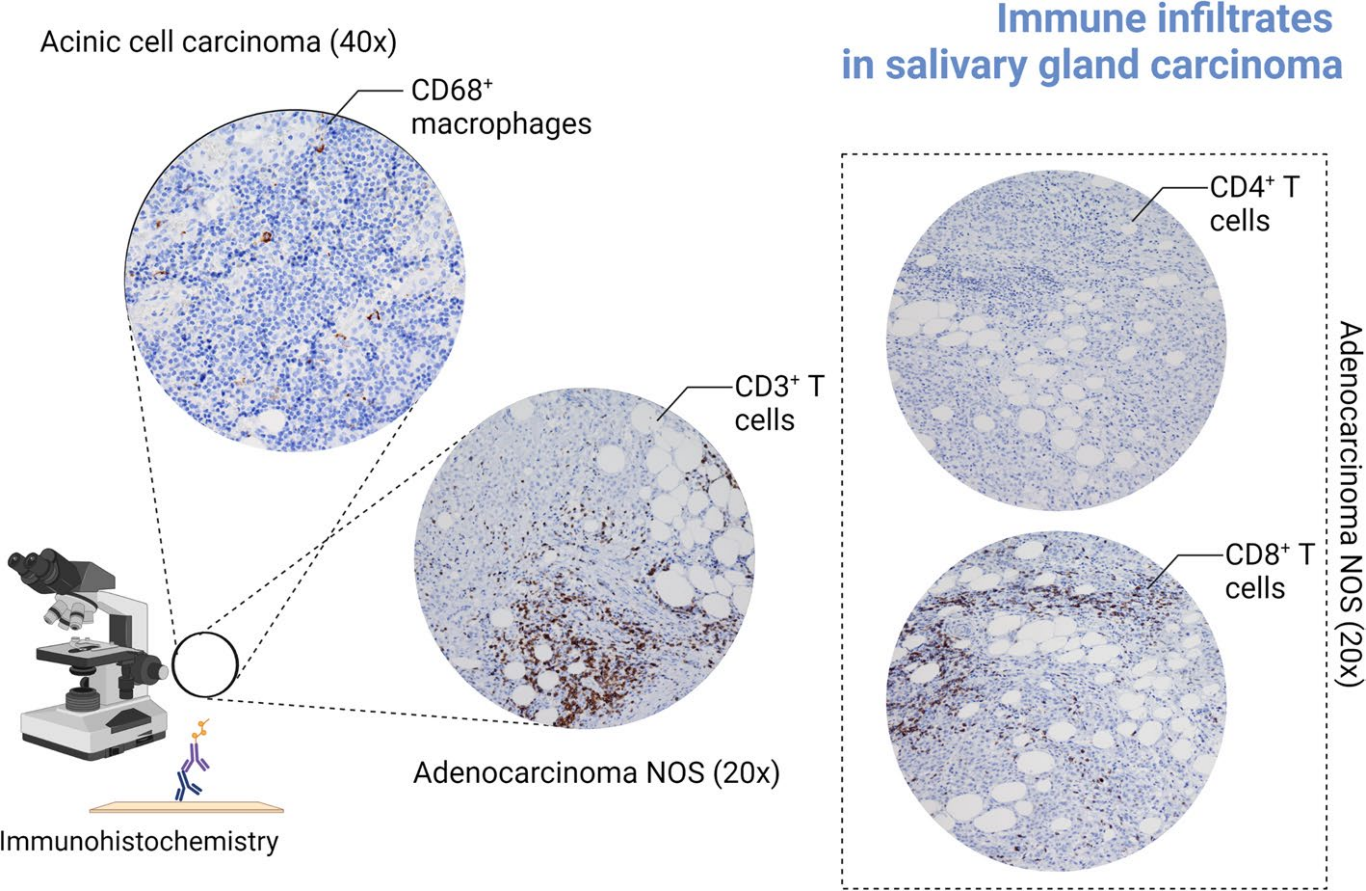
Immunohistochemical staining of CD68^+^, CD3^+^, CD4^+^ and CD8^+^ cell subsets. A subset of 10 cases was stained with CD3, CD4, CD8 and CD68 antibodies to specify the phenotypes of the infiltrating immune cells. In these selected cases, CD8^+^ T cells largely dominated the immune landscape of the TME and outnumbered CD68^+^ macrophages (Created with Biorender, No. FB24AJR43N)

Infiltration of tumors with TILs is associated with a better response to immunotherapy with immune checkpoint inhibitors (ICI) [37]. Therefore, a high expression of CD47 in tumor-infiltrating immune cells would protect these cells against macrophage-mediated phagocytosis and ensure a better response to ICI immunotherapy [17]. On the other hand, the expression of CD47 in TIICs could also label these cells as targets for anti-CD47 therapies [24]. In our study, subset analyses have revealed that the predominant immune cell subset in the TME of SGCs, is the CD3^+^ T cell subset. These CD3^+^ T cells were mostly of CD8^+^ phenotype and largely outnumbered the CD68+ macrophages (Fig. 3).

When evaluating the CD47 expression, our data showed that CD47 expression in TIIC is relatively high, but does not differ statistically from CD47 expression in tumor cells (data not shown). Furthermore, the proportion of CD47^+^ TIICs did not differ statistically between the histological subtypes of SGCs, Fig. 2b. The expression of CD47 in TIICs in the center of AdCC tumors could not be assessed due to the complete absence of TIICs in the tissue.

Next, we have correlated the proportions of CD47^+^ tumor cells in the center of the tumor and in the periphery of the tumor in each histological subtype. Surprisingly, no differences were observed in the proportions of CD47^+^ tumor cells clustered in the tumor center and the tumor periphery**,** nor in the proportions of CD47 + TIIC (data not shown). The data so far show that while CD47 expression varies between SGC subtypes, expression levels are similar between tumor cells and TIICs. Furthermore, the location of cells (either center of the tumor or periphery) had no effect on the CD47 expression.

### CD47 positive tumor cells are outnumbered by CD47 positive TIICs in mucoepidermoid carcinoma

The interplay between the tumor and the immune system extends beyond the tumor microenvironment (TME) [24]. To understand the mechanisms that drive the patient’s response/resistance to immunotherapy, the phenotypical signatures of both tumor cells and TIIC were analyzed [38]. To provide a broader view of the role of CD47 in the interplay of the tumor-immune system, we compared the proportions of CD47^+^ tumor cells and CD47^+^ TIICs in each histological subtype, and also in the central and peripheral tissue compartment. For each type of cancer, MEC, AdCC, ACC, SDC, and AdCaNOS, we have examined the center of the tumor and the periphery of the tumor to understand which CD47^+^ cells dominate the tissue compartment.

Initially, we did not observe differences in the tumor center of MEC, AdCC, ACC, SDC, and AdCaNOS, supplementary Figure 2. For each histological subtype, the proportion of CD47^+^ tumor cells in the tumor center was comparable to the proportion of CD47^+^ TIICs.

**Fig. 4:**
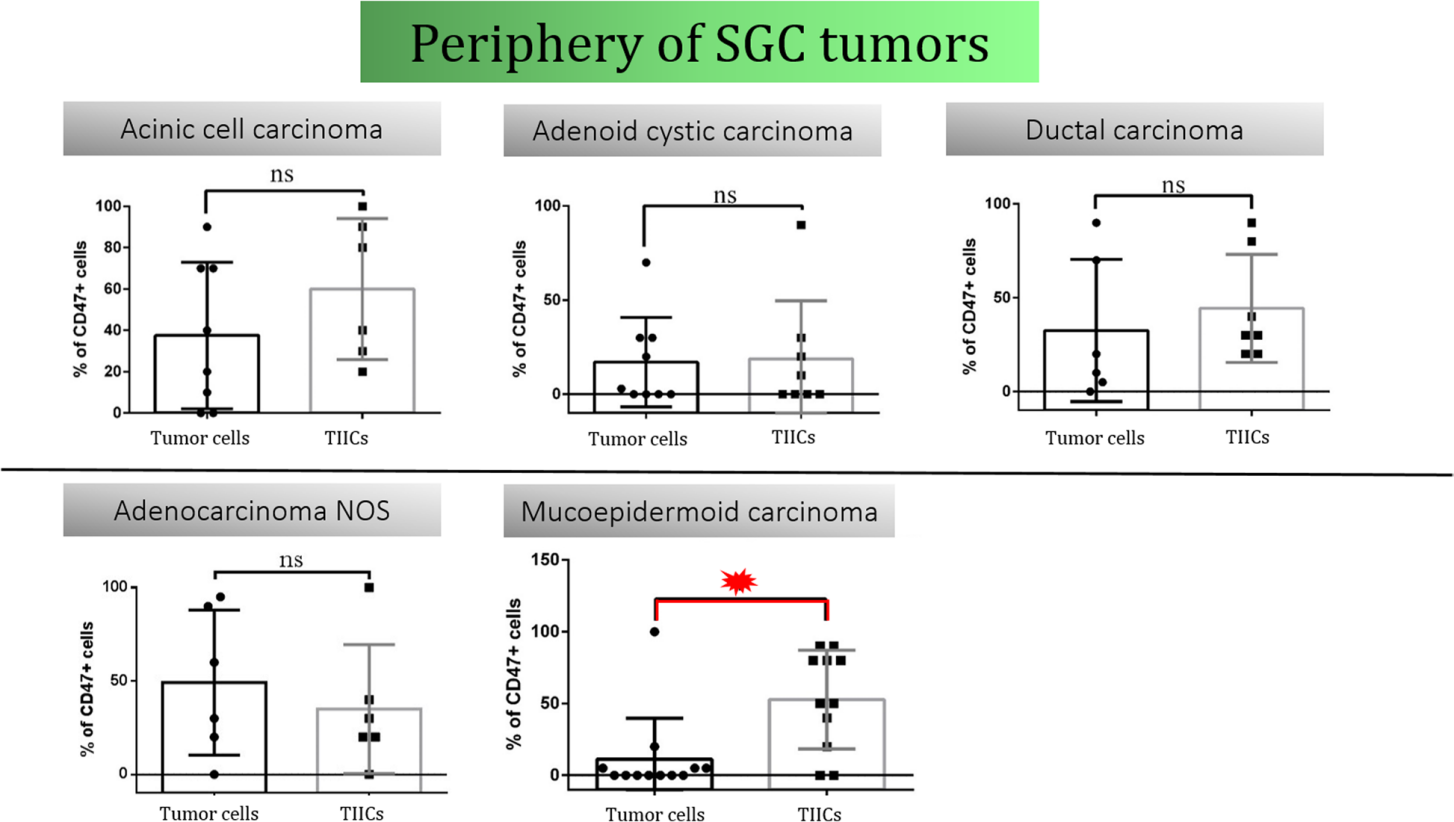
Periphery of SGC tumors. Thirteen patients with mucoepidermoid carcinoma (MEC), ten patients with adenoid cystic carcinoma (AdCC), eight patients with acinic cell carcinoma (ACC), six patients with salivary duct carcinoma (SDC) and six patients with adenocarcinoma not otherwise specified (AdCaNOS) were included. A significant difference in CD47 expression between tumor cells and TIICs was observed in the periphery of MEC tumors

However, we did observe a significant difference in the heterogeneity of CD47 expression between tumor cells and TIIC in the periphery of MEC, Fig. 4**.** This is particularly interesting as MEC is the most common SGC, but one of the subtypes with the fewest breakthroughs achieved so far, with the treatment strategy still primarily driven by tumor grade [28].

### Major differences in the CD47 expression on tumor cells and tumor-infiltrating immune cells were observed in the periphery of SGC tumors

Tumor stage and tumor grade are commonly used scoring systems to determine the prognosis of a patient [39]. According to the TNM classification system, stage I. SGCs are noninvasive tumors without lymph node involvement or distant metastases and stage IV. SGCs are invasive tumors with lymph node involvement and / or distant metastases [39]. Late stage cancers and poorly differentiated high-grade cancers are associated with significantly lower 5-year survival compared to early-stage and low-grade cancers [26]. In patients with SGC, the TNM grade and the tumor stage were described as the most relevant and predictive variables [26, 38, 40]. Other important prognostic factors include the patient’s age and condition, tumor site, radicality of the surgical procedure, and dose of radiation therapy [41].

In our next set of analyzes, we divided the 43 patients in our study cohort according to their tumor stages and tumor grades. As shown in supplementary Figure 3**,** tumor stage was not related to the proportions of CD47^+^ tumor cells in the center / peripheral of the tumor, nor to the proportions of CD47^+^ TIICs.

**Fig. 5:**
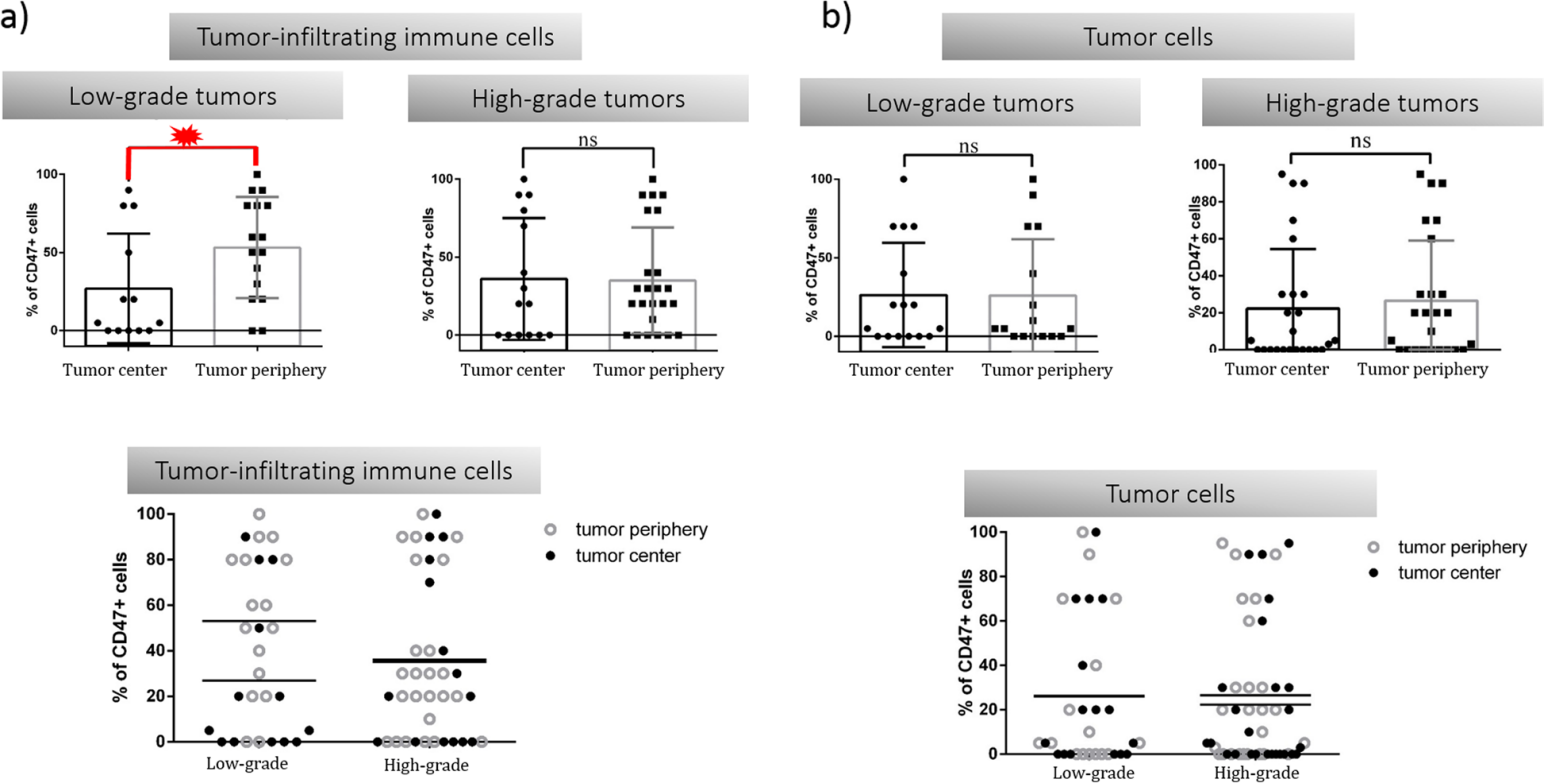
Differences between low-grade and high-grade tumors. Differences between low-grade and high-grade subgroups were not reflected in the CD47 expression status neither in TIICs, nor in tumor cells. In low-grade tumors, significantly higher expression of CD47 was observed in TIIC in the periphery of the tumor compared to TIIC in the center of the tumor

After dividing our patients into a low-grade and a high-grade subgroup, we did not observe significant changes in the proportions of CD47^+^ tumor cells between these groups, nor in the two different tissue compartments (tumor center, tumor periphery) within the same subgroup. However, in low-grade tumors, significantly higher expression of CD47 was observed in TIIC in the periphery of the tumor compared to TIIC in the center of the tumor, Fig. 5**.** Therefore, the data indicate that TIICs might be more efficiently protected against macrophage-mediated phagocytosis in the tumor periphery, while in the tumor center, TIICs are more susceptible to this type of elimination.

**Fig. 6:**
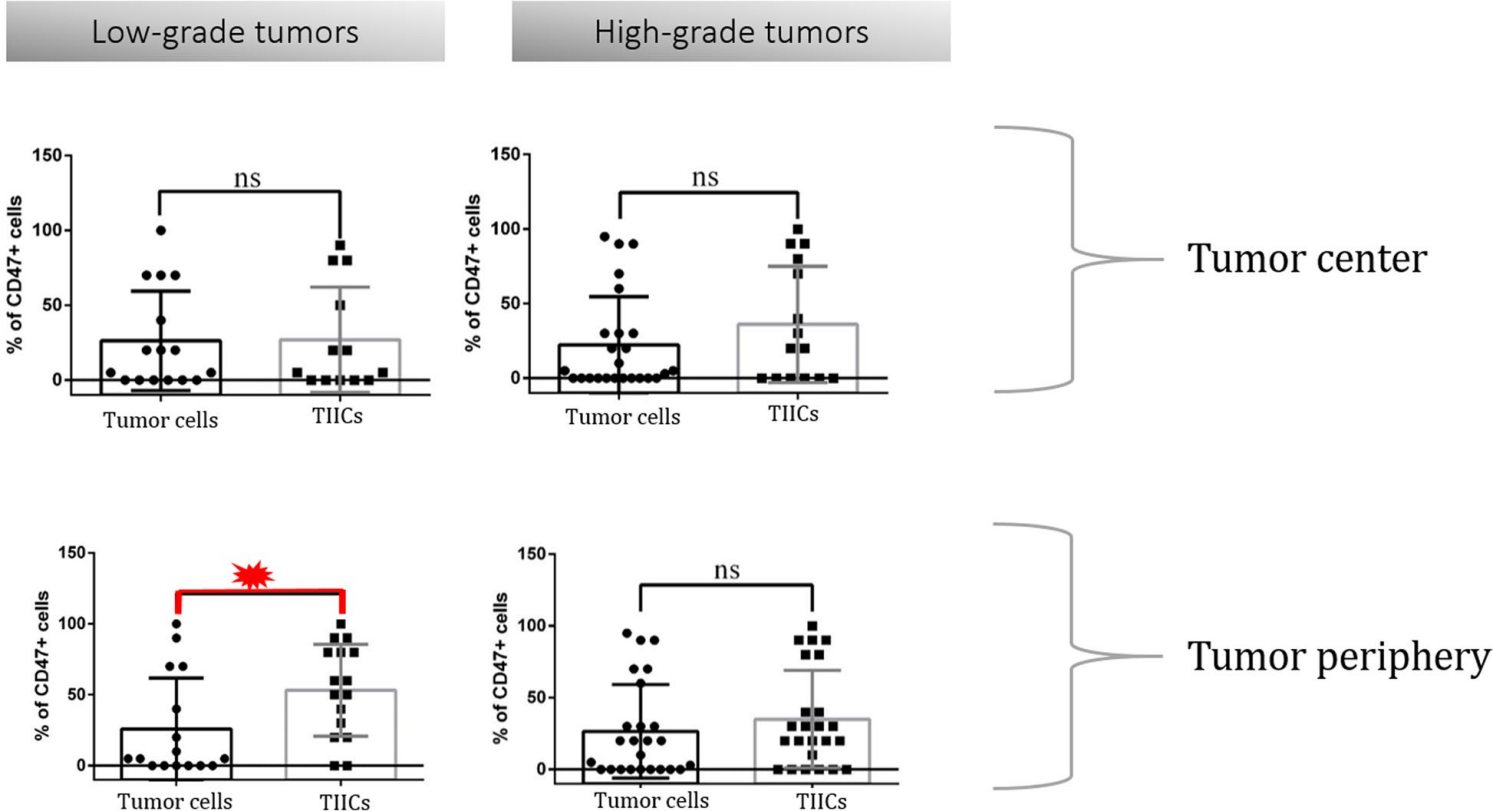
Differences between CD47^+^ TIICs and CD47^+^ tumor cells A detailed view on the CD47 expression in different cell types and tissue compartments. Low-grade tumors were characterized by a significantly higher CD47 expression in TIICs as compared to tumor cells. This phenomenon was observed in the tumor periphery

Since a higher proportion of CD47^+^ TIICs was observed in the periphery of low-grade tumors, we pursued the investigation of the tumor-immune system interaction in this tissue compartment. Interestingly, in low-grade tumors, we detected a significant difference in CD47 expression between tumor cells and TIIC in the periphery, with more than half of TIICs expressing CD47 as compared to tumor cells, Fig. 6. The data suggest an important role for CD47 in TIICs found in the periphery of the tumor. We hypothesize that an increase in CD47 expression on periphery TIICs could protect newly recruited leukocytes from macrophage-mediated phagocytosis, while a decrease in CD47 on tumor center TIICs could facilitate the removal of old or anergic leukocytes, allowing antigen-presenting cells to acquire more tumour antigens, ultimately sustaining the antitumor response.

## Discussion

SGCs are rare and extremely heterogenous tumors [26, 28]. Extensive surgery is currently the mainstay treatment for SGC [26]. However, treatment options in recurrent metastatic disease are quite limited [26, 28]. Radiotherapy may support local control of the disease; however, in unresectable tumors, only 20% of patients with advanced-stage disease achieve disease remission with radiotherapy as a single treatment [42]. Immunotherapy with immune checkpoint inhibitors currently dominates the landscape of oncologic treatment in metastatic diseases [43]. Anti-PD-1 therapies have shown promising results in early clinical trials, but only histologically driven approaches can reveal tumor resistance/sensitivity to immunotherapy [44]. This stems from the fact that the TIIC content in SGCs was shown to differ among histological subtypes [38, 45].

Anti-CD47 therapy is currently being evaluated in clinical trials [14, 21]. Its efficacy depends on the ability of anti-CD47 mAbs to increase the tumor cell phagocytosis and prime antitumor CD8^+^ T cell responses [20]. Furthermore, CD47 was shown to enhance the recruitment of CD8^+^ T cells in a fibrosarcoma model suggesting a complex regulatory function of CD47 in the TME [46].

CD47 overexpression has been described in several types of cancer and, thus, may be predictive of treatment response [22]. Although anti-CD47 immunotherapy could also be beneficial in SGCs, the expression of CD47 on the cell surface of TIICs could, on the other hand, affect immune infiltrates in TME and worsen the prognosis of patients [24].

The impact of anti-CD47 immunotherapy on both the NK cells and T cells remains to be elucidated, particularly in SGCs. To date, anti-CD47 treatment has been already shown to elicit a positive effect on intratumoral NK cell activity, and furthermore, a study by Kim et al. suggests that blocking of CD47 activates NK cell-mediated lysis of head and neck squamous cell carcinoma cell lines [15, 47]. However, to date, a detailed analysis of CD47 expression in both tumor cells and TIICs has been missing. In this study, we performed an IHC analysis of CD47 expression in both tumor cells and immune cells. Moreover, to understand the immune context of the SGC TME, we have separately analyzed both the tumor center and the tumor periphery.

We observed that the highest proportions of CD47^+^ tumor cells were in the tumor periphery of AdCaNOS. These results were in contrast to MEC where CD47^+^ tumor cells were poorly presented and, moreover, accompanied by a high load of CD47^+^ TIICs.

To investigate how the biological behavior of SGC tumors is affected by CD47 expression, we have also correlated the expression status with the grade and stage of the tumor. Low-grade SGCs had more profound differences in CD47 expression in TME. CD47^+^ TIICs were significantly higher in the tumor periphery compared to the tumor center. Also, when looking closer to the peripheral compartment of the SGC TME, we found significantly higher proportions of CD47^+^ TIIC compared to CD47^+^ tumor cells. This could by all means significantly affect the clinical efficacy of anti-CD47 immunotherapy.

## Conclusion

Although the reason for the higher expression of ‘don’t eat me’ signals in TIICs in the tumor periphery and a reduction in the tumor center is unclear, we hypothesize that it could be a mechanism to protect newly recruited leukocytes from macrophage-mediated phagocytosis, while also allowing the removal of old or exhausted leukocytes in the tumor center. However, the data suggest that macrophage-mediated phagocytosis in SGCs occurs mostly in the tumor periphery and predominantly affects TIICs. As a result, anti-CD47 therapy for SGC might not be a ‘silver bullet’, as many TIICs would also be affected, especially in MEC tumors where CD47 is more expressed in TIICs than in tumor cells themselves. On the other hand, previous studies have shown an additive effect of anti-CD47 treatment when administered in a combination therapy with radiation. Thus, combinatorial treatment approaches utilizing anti-CD47 with other therapeutic modalities may still be promising [46].

## Supplementary Information

**Additional file 1:** Supplementary Figures 1, 2 and 3.

## Data Availability

The authors are happy to share data on request to the corresponding author.
