# Supplementary material for: The expression profiles of CD47 in the tumor microenvironment of salivary gland cancers: a next step in histology-driven immunotherapy
Source: BMC Cancer. 2022 Sep 28;22:1021. doi: 10.1186/s12885-022-10114-4 (PMC9520840; doi:10.1186/s12885-022-10114-4)
Supplement: Supplementary file 1 — Additional file 1: Supplementary Figures 1, 2 and 3. [file 12885_2022_10114_MOESM1_ESM.docx]

SUPPLEMENTARY FIGURES


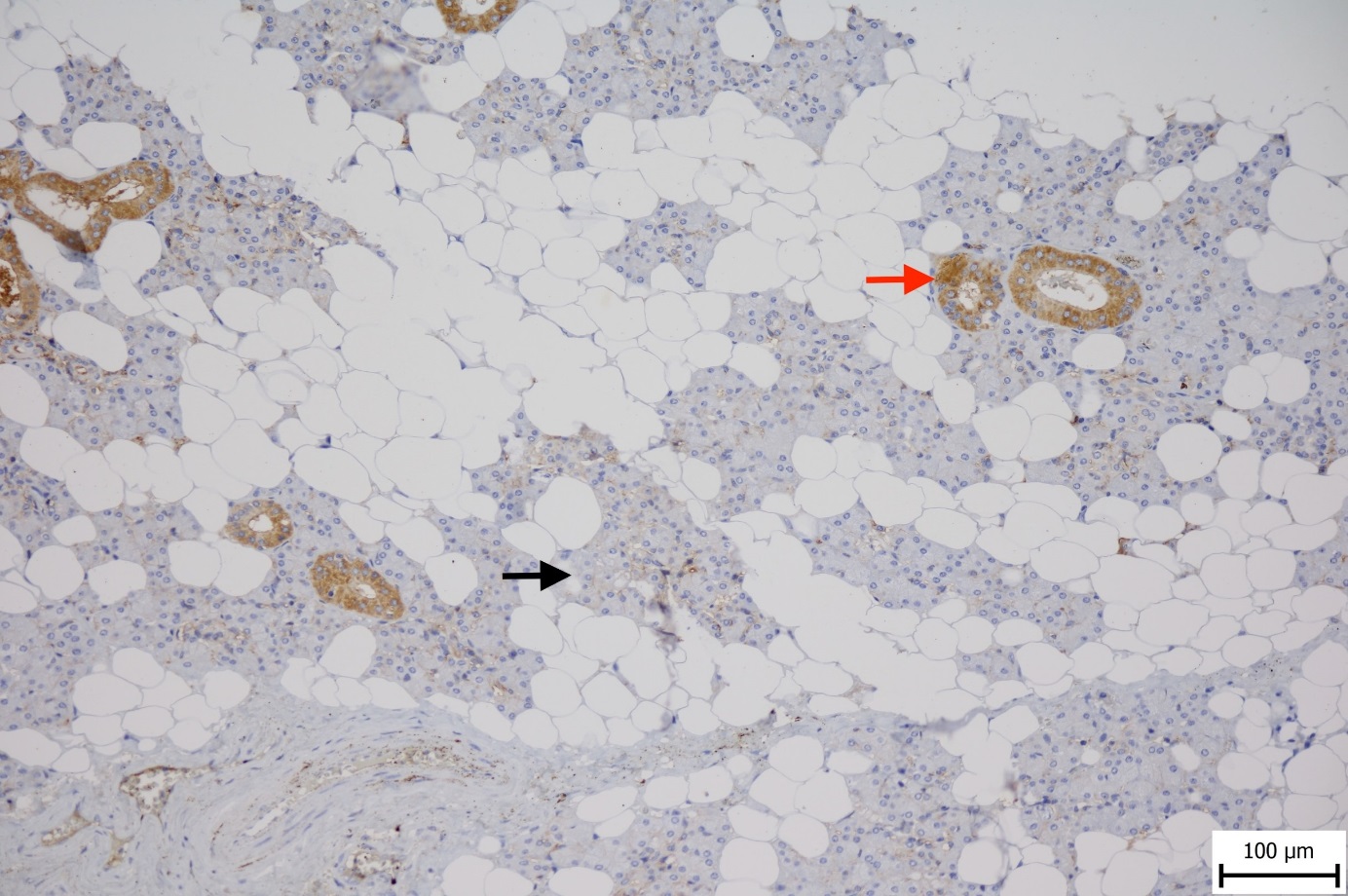


**Supplementary Figure 1. Expression of CD47 in the adjacent normal tissue**

Immunohistochemistry (IHC) of CD47 expression in adjacent salivary gland tissue. Representative image shows negative CD47 expression in acinic cells (black arrow) and positive cytoplasmatic and membranous staining in ductal epithelium (red arrow).


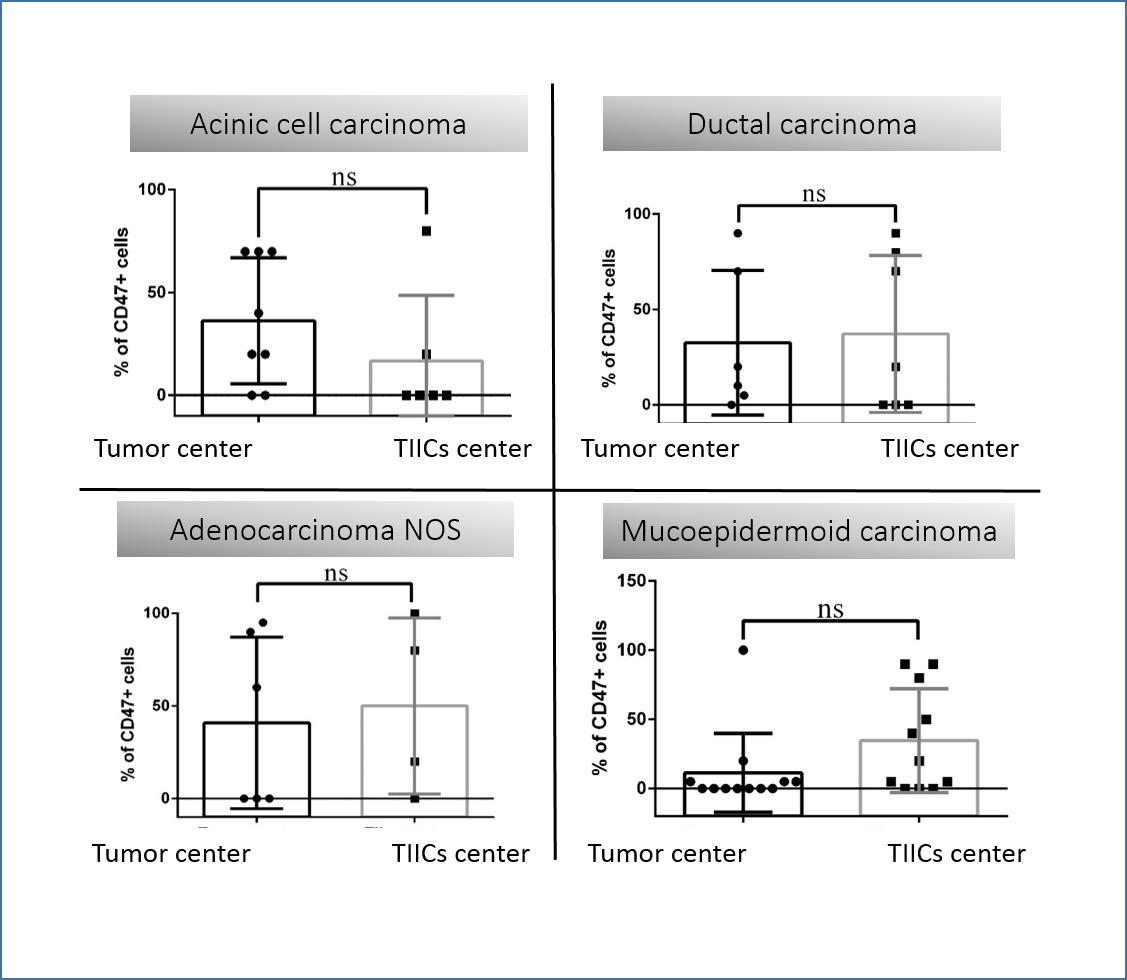


**Supplementary Figure 2. Expression of CD47 molecule in the tumor center.**

No differences were observed in the tumor center of MEC, AdCC, ACC, SDC, and AdCaNOS. For each histological subtype, the proportion of CD47^+^ tumor cells in the tumor center was comparable to the proportion of CD47^+^ TIICs.


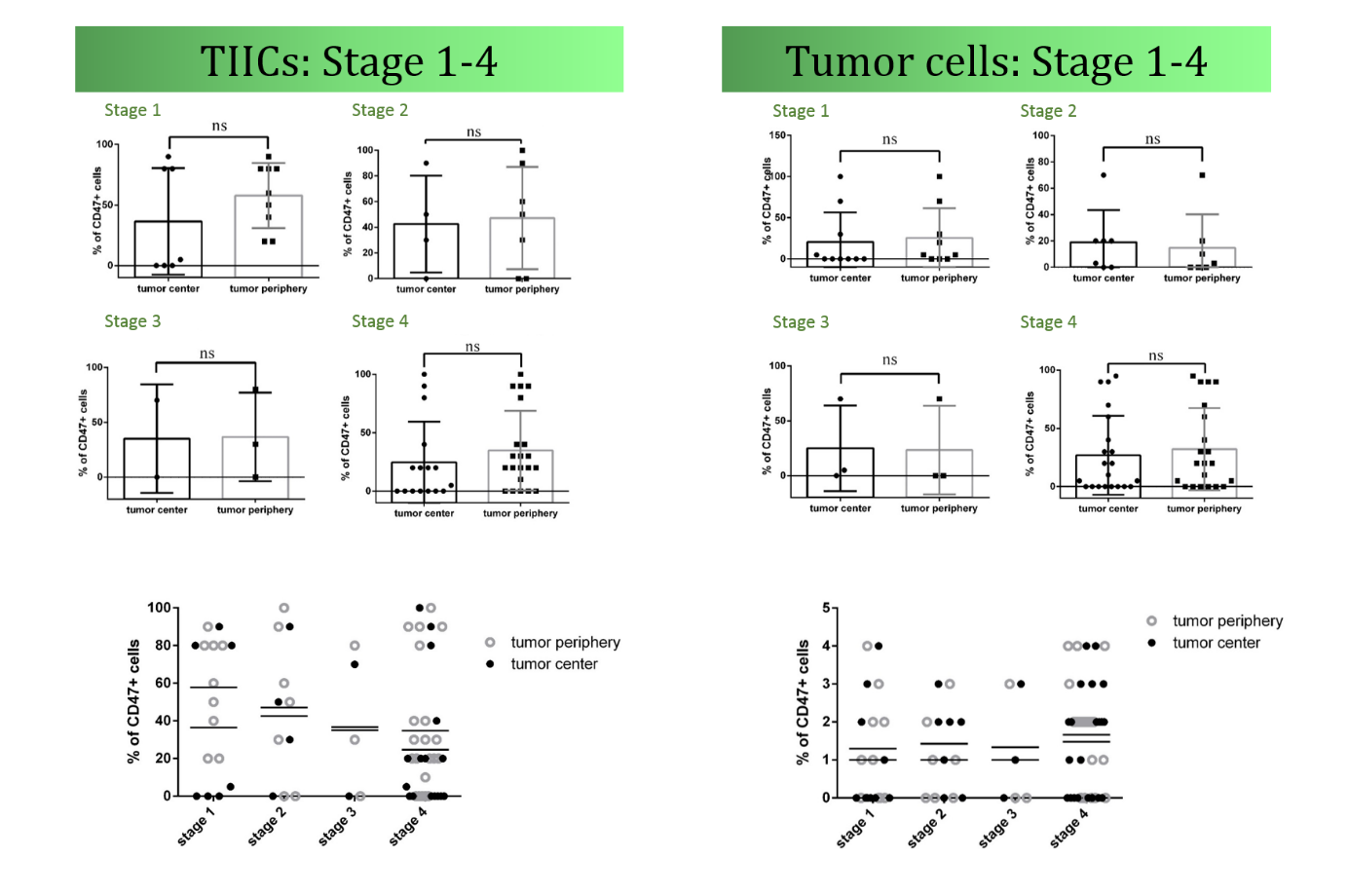


**Supplementary Figure 3. Tumor stage does not affect CD47 expression**

Tumor stage was not related to the proportions of CD47^+^ tumor cells in the center / peripheral of the tumor, nor to the proportions of CD47^+^ TIICs.
